# Community pharmacies as a place for informal carer support in mental health and wellbeing

**DOI:** 10.1007/s11096-023-01606-9

**Published:** 2023-06-03

**Authors:** Charlotte Lucy Richardson, David Black, Laura Lindsey, Hamde Nazar

**Affiliations:** Faculty of Medical Sciences, School of Pharmacy, Newcastle University, King George VI Building, Newcastle Upon Tyne, NE2 7RU UK

**Keywords:** Carers, Community pharmacy, Mental health and wellbeing, Social prescribing

## Abstract

There are 5.3 million informal carers in the United Kingdom who take on caring responsibilities for family and friends. Informal carers can be forgotten patients within health and care services, yet because of carer burden, they are at risk of deterioration in health and wellbeing. There are higher levels of anxiety, depression, burnout and low self-esteem amongst carers but, to our knowledge work to date has mainly focused on supporting carers to provide better care for their family member, and less on carers’ health and wellbeing. There is increasing interest in social prescribing as a method of linking patients with community-based services to improve health and wellbeing. Initiatives have included social prescribing via community pharmacies which are already recognized to be accessible for support and signposting. The coming together of community pharmacy services and social prescribing could represent a framework to better support carers in their mental health and wellbeing.

## Introduction

In the United Kingdom (UK) there are currently 5.3 million informal carers and estimated to be 20 million across Europe [1], and this is likely to increase with time due to the ageing population [2, 3]. An informal carer is defined as someone who looks after a friend or family member who, because of their medical needs (including disability, mental health and frailty), requires help and cannot manage without the person’s support; this care is unpaid[4].

The European Commission has recognized the legal status of carers as one of the cornerstones of health and long-term care systems across Europe [5, 6], but policy developments and initiatives are uneven across Europe [7]. Those countries which have recognized the legal status of carers have most often interpreted this in terms of flexible employment arrangements to better allow for caring responsibilities [8]. It should be noted that the term ‘informal carer’ is the most used term within literature however, some carers can find this term to be misrepresentative as it reduces the importance and impact of their role [9]. ‘Informal carer’, or simply ‘carer’ are used throughout this commentary in line with the current preferred terminology, and to refer to informal, unpaid carers as opposed to formal or paid carers.

The majority of informal carers provide care to family members with the most common arrangement being adult children providing support to parents who do not live with them [2]. The type of care that informal carers provide varies substantially and can include support with getting to appointments; medicines such as ordering, collecting and helping with administration; help with everyday tasks such as getting out of bed, and personal care such as bathing and feeding; and emotional support such as helping someone cope with disease symptoms [4]. Most often these tasks are taken on without training or detailed instructions.

The aim of this commentary is to provide insight into how informal carers are often the forgotten patients within care services, yet because of their carer burden, they are at greater risk of deterioration in their mental health and wellbeing, which can manifest as caregiver burnout, anxiety, and depression.

## Carer identification

Carers can struggle to self-identify themselves as such [10]. It is reported that self-identification as a carer can occur over a prolonged period of time with 51% of carers taking one year and 36% taking over three years to identify with their role as a carer [11].

One of the reasons that people may not self-identify as carers is the sense of obligation to care. This is where someone feels morally responsible for looking after and caring for someone, usually a friend or family member [12]. Obligation to care is caused by intra-familial bonds where some carers view their caring relationship as a ‘normal’ familial relationship [13]. It can also arise from a feeling of guilt or moral debt and is more common in collectivist cultures, such as in East Asian countries compared to individualist ones, such as the UK [14, 15]. Obligation to care means people are less likely to reach out for help, since they do not necessarily identify as carers [12].

Better identification of carers may be one way of improving the support they receive. However, carer identity is complex and not a discrete state of being. A study looking at carer identity in patients with early cognitive impairment as opposed to diagnosed dementia, found carers were unsure about the norms associated with being a carer and they did not see themselves as carers as the current needs of their loved one were not severe enough to see themselves in this role [16]. For this reason, there is an argument that not every carer wants to, or will, identify as a carer and as such we should reconsider encouraging people to develop a traditional carer identity as the only way to facilitate better support for them [17, 18].

Equally, carers are not always recognized as such by healthcare professionals. This limits the signposting and support they are offered within practice settings [19, 20]. If healthcare professionals were better prepared to identify and support informal carers, this could help carers to recognize the significance of their caring status particularly for their own health and wellbeing regardless of whether they identify fully with the term carer or not [21].

## Carer burden, and mental health and wellbeing

It is recognized that the health and wellbeing of patients and their carers are inextricably linked, meaning if we look after one we inadvertently look after the other [22]. Poor outcomes for the patient can lead to a substantial health and wellbeing risk to the carer, who may themselves go on to need care and vice versa [23, 24].

Several surveys and reports have highlighted the impact of caring on carer health and wellbeing with higher levels of anxiety and depression amongst carers than those without caring responsibilities, as well as a more limited ability to engage with appointments for their own health due to their caring responsibilities [25–27]. This is likely to have worsened significantly since the pandemic caused by Coronavirus disease in 2019, where it is estimated that 4% of adults in the UK took on caring roles which they were not doing before [25].

Mental and physical health problems are common amongst carers due to the emotional exhaustion of caring and the physical tasks involved [28]. Carers commonly experience burnout and one study found that 20% of carers also experienced low self-esteem [28, 29].

For carers, mental wellbeing is influenced by their caring burden [12]. Carer burden is caused by the direct stress and anxiety of caring coupled with often little time to themselves to do things they enjoy [30]. The *Commitment to Carers* report highlighted the significance of this with a concluding theme being the need to *“recognise that I also may need help both in my caring role and in maintaining my own health and wellbeing”*[4][pg.11]. Consequently, principle 8 of the National Health Service (NHS) in England’s Commission for Carers is: prioritise carers’ health and wellbeing. Additionally, in 2022 the National Institute for Health and Care Research encouraged researchers to consider the role of informal carers’ health and wellbeing as a priority for research [31].

As previously mentioned, healthcare professionals struggle to identify carers and this is recognized by the carers themselves who feel unseen within health settings [32]. A majority of carers (66%) feel that signposting to supportive services by healthcare professionals is limited and that it is more likely to come from charities and other carer organizations than practicing healthcare professionals [33].

Much of the work to date has focused on supporting and empowering carers to provide better care, rather than looking after their own health and wellbeing as a carer [34–36], for example, through supporting carers in better medicines management [36–38]. There is a potential change in mindset needed for healthcare professionals who currently focus more on patients as individuals rather than together with their carers, where carers also have health and wellbeing needs. As such professionals may need to consider a more holistic care approach when supporting carers. This is in line with recommnedations from Carers UK in 2022 which highlighted the need for training healthcare professionals to “*identify, signpost and support carers when they encounter them, particularly in relation to carers’ health and wellbeing”*[11][Pg. 32]. This is particularly important as caring is increasingly recognized as a social determinant of health potentially contributing to health inequalities [11].

## Social prescribing for carers

In recent years, there has been a growing interest in the role of social prescibing [39]. Social prescribing refers to a process of linking patients in primary care with community-based sources to help improve their health and wellbeing, such as practical information and advice, community activity, physical activities, befriending and enabling services [40].

In 2019 Drinkwater et al., recommended social prescribing for *“patients who may require a greater level of social and emotional support to improve wellbeing and health than is available in routine care”*[41][pg.2]. Yet in a recent systematic review of social prescribing interventions across care settings only two of 16 studies explictly made reference to including carers in services [39, 42, 43]. Conversley, Hamilton-West recognized the potential role of social prescribing as a way of supporting carer mental health and wellbeing and as such there could be more emphasis on social prescribing initiatives focusing on carers [44].

## The role of pharmacy in supporting mental health and wellbeing

Community pharmacy services are recognized to be accessible and convenient, especially given appointments are not necessary and increases in pharmacist prescribers [37, 45, 46]. Pharmacies are also increasingly recognized spaces for community-based mental health support, with examples being the screening and supportive management of depression and pain [47, 48, 49]. This is also true of social prescribing where there have been interventions based-in or involving community pharmacies, several of which aimed to address issues relating to mental health and wellbeing [50–52].

One study which aimed to advance mental health support in pharmacies, noted that the patients' priority area of concern in relation to their mental health was caring responsibilities on four occasions (76 patients enrolled in the study who each had up to three consultations)[53]. Using this literature, it is not unreasonable to suggest that pharmacies are well placed to help identify and support those at risk of deterioration of their own mental health and wellbeing stemming from caring responsibilities. Although, it should be noted, that of the interventions to date, funding and renumeration for services have been variable and this may be a barrier moving forward to establishing impactful carer services.

## Pharmacy, carers, and mental health and wellbeing

Many carers regularly visit pharmacies to collect their loved-one’s medicines and therefore may have an existing relationship with pharmacy staff [38]. Pharmacies are already a place for *ad-hoc* signposting of carers to useful services and groups, for example, mental health services, social care, support groups and charities [54–56]. In Australia, the work of McMillan et al., provides an example of a more substantial carer service [56]. The authors concluded pharmacy staff are well positioned to support carers, through meeting them and co-developing an action plan, although no evaluation of carer health or wellbeing appears to have been reported but was part of the service [56].

One social prescribing evaluation did consider carers as service users and included referal pathways via a pharmacy, however this was not the main aim of the service which was targetted at the public more broadly [42]. A further study on carers of patients with Alzheimer’s Disease went as far as saying that pharmacists are the most accessible health care professionals to provide carer advice about disease mangement which could contribute to easing the carer burden which can manifest as anxiety and depression. Again, this was not the aim of the service and as such mental health and wellbeing were not measured as an outcome [57].

We postulate that the coming together of community pharmacy services and social prescribing could represent a framework to provide better support for carers for their mental health and wellbeing. The design and specificity of any pharmacy-based carer services needs to be founded on evidence but could include pharmacies as a place for the identification, signposting and/or prescribing for carers. We are suggesting that interventions should not solely be directly aimed at improving carers’ ability to care, but there should also be interventions in which the carers own mental health and wellbeing is the priority of the intervention although, it is possible that by improving carer health and wellbeing a consequence could be to also improve carer ability to care and indirectly patient outcomes.

## Conclusion

Informal carers are a forgotten group within healthcare with unique care needs of their own. They are particularly at risk of deterioration in mental health and wellbeing due to the burden associated with caring roles. It is possible that pharmacy has a role to play in delivering services that aim to support, identify, and care for carers. We advocate for development of services which consider how carers’ mental health and wellbeing can be supported within pharmacy settings possibly through social prescribing. Yet considerable work is needed to understand more clearly the inextricable link between patient and carer health and wellbeing.
